# Supplementary material for: Mosquitoes Possess Specialized Cuticular Proteins That Are Evolutionarily Related to the Elastic Protein Resilin
Source: Insects. 2023 Dec 11;14(12):941. doi: 10.3390/insects14120941 (PMC10743668; doi:10.3390/insects14120941)
Supplement: Supplementary file 1 [file insects-14-00941-s001.zip › FigS1-R&R-alignment.pdf]

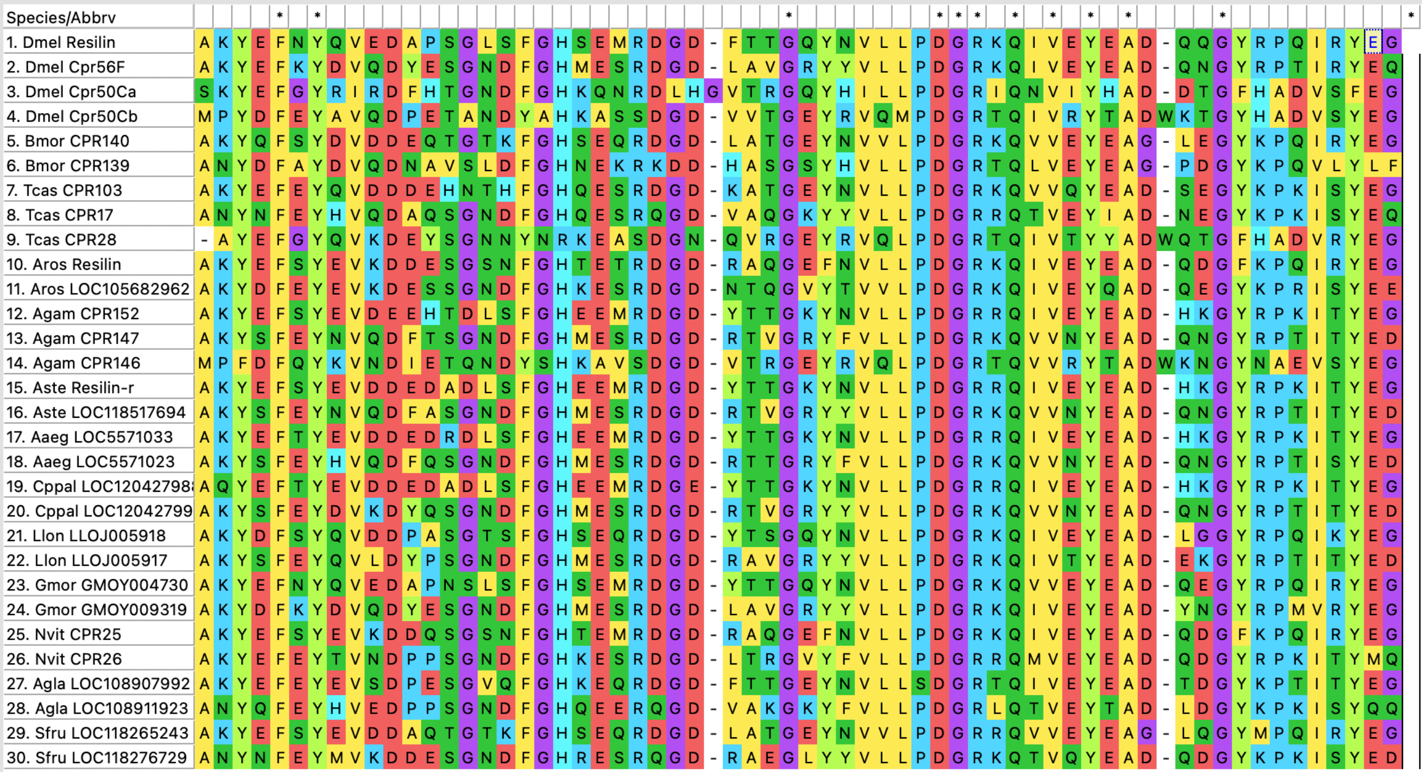

Figure S1. Alignment of the R&R Consensus of resilin homologous proteins.

The amino acid sequences of R&R Consensus were aligned by ClustalW implemented in MEGA11. The used sequences and their nomenclature are same as Figure 1.
