# Supplementary material for: Mosquitoes Possess Specialized Cuticular Proteins That Are Evolutionarily Related to the Elastic Protein Resilin
Source: Insects. 2023 Dec 11;14(12):941. doi: 10.3390/insects14120941 (PMC10743668; doi:10.3390/insects14120941)
Supplement: Supplementary file 1 [file insects-14-00941-s001.zip › FigS2-ChitinBindingAssay.pdf]

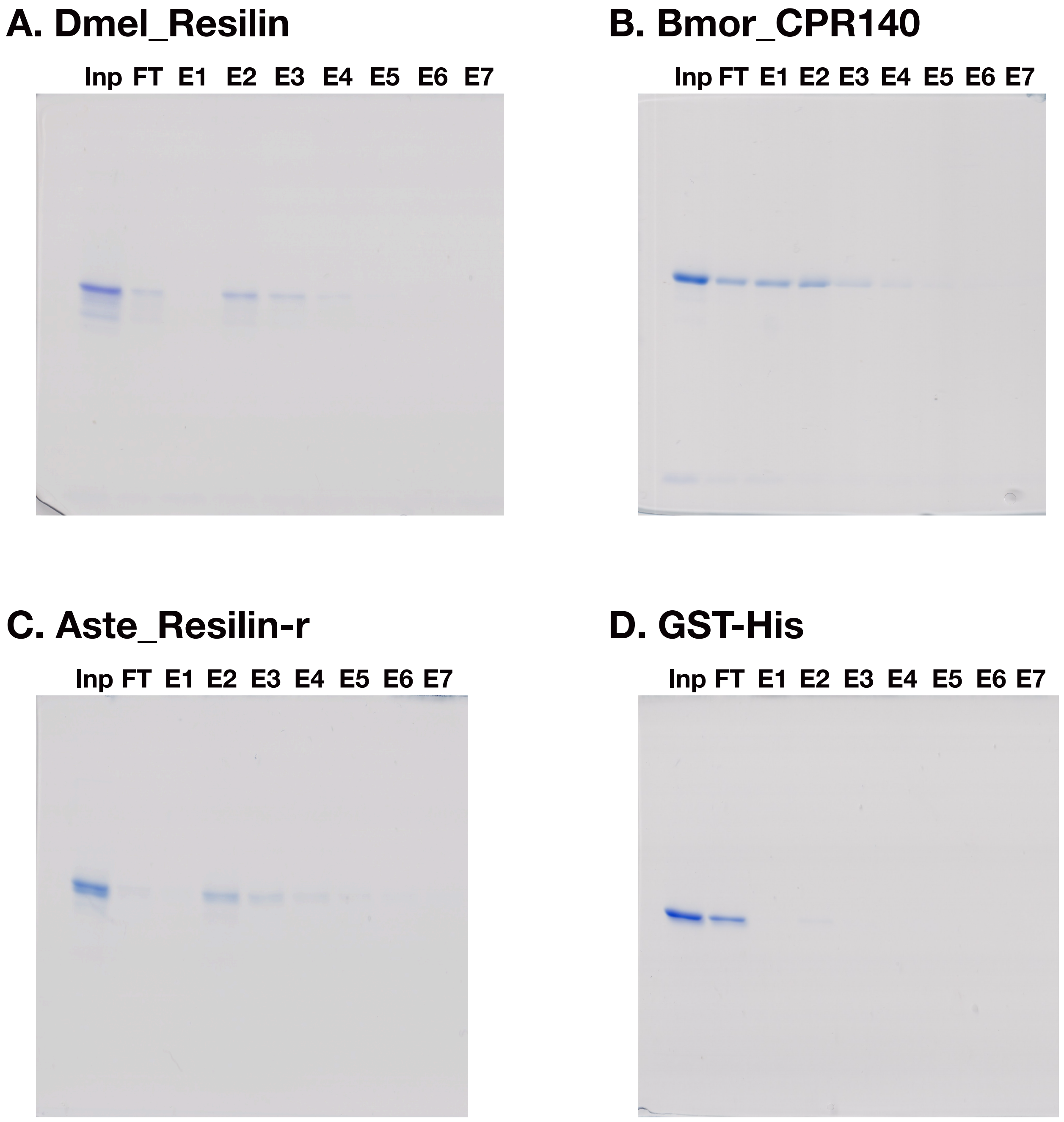

Figure S2. Chitin-binding assay of recombinant proteins.

Two hundred μg of purified recombinant proteins for Dmel_Resilin **(A)**, Bmor_CPR140 **(B)**, and Aste_Resilin-r **(C)** in 200 μl of chitin-binding buffer were loaded onto a 200-μl chitin column. The flow-through was loaded onto the same column four more times. After the column was washed well, the bound proteins were eluted with 200 μl of 8 M urea in binding buffer 7 times. The recombinant protein without resilin fragment was also examined as a negative control **(D)**. Aliquots of protein input (*Inp*), the final flow-through (*FT*), and the 1st – 7th elution fraction (*E1-7*) were analyzed by SDS - polyacrylamide gel electrophoresis.
